# Adsorption Geometry of Alizarin on Silver Nanoparticles: A Computational and Spectroscopic Study

**DOI:** 10.3390/nano11040860

**Published:** 2021-03-27

**Authors:** Cristina Gellini, Marina Macchiagodena, Marco Pagliai

**Affiliations:** Dipartimento di Chimica “Ugo Schiff”, Università degli Studi di Firenze, via della Lastruccia 3–13, 50019 Sesto Fiorentino, Italy; marina.macchiagodena@unifi.it

**Keywords:** alizarin, Raman spectroscopy, SERS, silver nanoparticles, DFT calculations

## Abstract

The knowledge of the adsorption geometry of an analyte on a metal substrate employed in surface enhanced Raman scattering (SERS) spectroscopy is important information for the correct interpretation of experimental data. The adsorption geometry of alizarin on silver nanoparticles was studied through ab initio calculations in the framework of density functional theory (DFT) by modeling alizarin taking into account all the different charged species present in solution as a function of pH. The calculations allowed a faithful reproduction of the measured SERS spectra and to elucidate the adsorption geometry of this dye on the silver substrate.

## 1. Introduction

Anthraquinone derivatives are chemical species used as dyes or lakes in textiles and paintings from ancient Egyptians until today [1,2,3,4,5,6]. These substances can be easily obtained from plants, such as alizarin and purpurin extracted from rubia tinctorum, or insects, such as carminic and kermesic acids obtained from cochineal. Art historians, curators, researchers, and restorers have determined the presence of these substances in works of art and cultural heritage by means of various techniques [2,5,7,8,9], including Raman spectroscopic analysis [9,10,11,12,13,14,15]. Among these organic dyes, one of the first anthraquinone derivatives used in painting is alizarin, a molecule which confers a red color with a blue undertone. Its vibrational properties have been studied by several authors [16,17,18,19,20,21,22], by means of Raman spectroscopy. The experimental spectra have been also assisted by density functional theory (DFT) calculations for the assignment of the vibrational modes and for modeling the molecular geometry [16,18,21]. Since Raman spectroscopy suffers from low sensitivity and the spectra of organic dyes could be worsened by the fluorescence background [10,23], surface enhanced Raman scattering (SERS) spectroscopy has been revealed as a useful technique to overcome these problems [16,24]. This is one of the reasons SERS spectroscopy is today applied to investigate samples of interest in chemistry, biology, medicine, cultural heritage, and other sectors [25,26,27,28,29,30,31].

SERS spectroscopy relys on the interaction between the molecule of interest (specially through heteroatoms such as N or O) and an appropriate substrate, to obtain a huge enhancement of the Raman signals. The molecule adsorbs on the substrate surface, which must contain high-reflective metals, such as silver, gold, or copper. The strong localization of the electromagnetic field associated with the collective excitation waves, usually called plasmons, of the electrons near the nanostructured metal surface, allows obtaining enhancements of several orders of magnitude (usually a factor up to 10${}^{6}$) for the Raman signals of the adsorbed molecules. In addition to this mechanism, a chemical enhancement contribution, which usually provides Raman enhancement factor up to 10${}^{2}$, can occur, which is essentially due to a charge transfer (CT) process between the adsorbed molecules and the metal substrates [32,33]. The formation of chemical interaction between the molecule and the active sites of the metal surface is also responsible for the perturbation of the molecular polarizability, and therefore of vibrational frequency shifts, which provide useful information on the adsorption geometry of the molecule on the metal substrate. [25,26,27,28,29,30,31].

Despite the large volume of experimental and theoretical data on alizarin (AZ), some questions are still open regarding the adsorption geometries on nanostructured silver substrates [10,12,17,18,19,22,24,34,35,36,37], especially in relation with the existence of different ionic species in solutions.

Therefore, in the present study, we re-analyzed the SERS spectra of AZ interacting with silver nanoparticles (AgNPs), by considering all the species present in solution at pH between 10 and 11. The adsorption of the different AZ species in solution on AgNPs determines the SERS spectral features, which have not been considered in detail in previous studies. The interpretation of the experimental findings was accomplished by performing DFT calculations, which represent a useful support for the interpretation of the SERS spectra [17,18,19,22].

Among the different metals, silver is one of the most computationally studied species. In particular, it has been observed that DFT calculations allow a correct determination of both the shift of the vibrational frequencies and the relative intensities when the analyte is adsorbed on a nanostructured surface modeled as Ag${}^{+}$ or small charged silver clusters. This results should not be a surprise, because it has been experimentally ascertained that Ag(I) is present on nanostructured surfaces [38,39].

Moreover, it has been observed [40,41] that, in the first step of silver reduction, the formation of both (Ag${}_{3}$)${}^{+}$ and (Ag${}_{4}$)${}^{2+}$ occurs; consequently, for the first time, DFT calculations on the AZ${}^{-}$ (monoanionic) and AZ${}^{2-}$ (dianionic) species interacting with these clusters were carried out for the first time. It has been observed that DFT calculations performed on model systems made up by the adsorbate bound to a single Ag${}^{+}$ or small charged cluster are suitable to simulate the active sites present on silver nanostructured surfaces [25,39,42,43,44,45,46,47,48,48]. This approach has been revealed able to faithfully reproduce the SERS spectral features of several systems [38,39,42,43,47,49,50,51,52,53,54], regarding both frequency positions and relative intensities, and to provide information on the chemical adsorption geometry. Therefore, the calculations in the present study aimed to model the adsorption geometry on silver nanoparticles of the different dye species in solution and to reproduce the measured SERS spectra.

## 2. Materials and Methods

### 2.1. Silver Nanoparticles Synthesis and Chemicals

The AgNPs were prepared by reducing AgNO${}_{3}$ (99.9999% purity, Aldrich, Germany) with NH${}_{2}$OH-HCl (99.9% purity, Aldrich) in extra-pure distilled water (HPLC grade, Lichrosolv, Merck ) according to Leopold and Lendl procedure [55]. The pH value of the colloidal suspension was 10–11. AgNPs were remarkably stable, conserving their distinctive surface plasmon band centered at 409 nm for several weeks. AgNPs were activated before use by adding LiCl (>99% purity, Aldrich) 1 M aqueous solution [56,57] to a final concentration of 5 × 10${}^{-6}$ M (10 μL in 2 mL AgNPs dispersion). Alizarin (>99% purity, Aldrich) with 10${}^{-3}$ methanol (>99% purity, Merck) stock solution was added to the colloidal dispersion to reach the final concentrations of 2.5 × 10${}^{-6}$ and 1.2 × 10${}^{-5}$ M.

### 2.2. Instruments

Extinction spectra were recorded with a Cary60 UV-vis-NIR spectrophotometer (Agilent Technologies, S. Clara, CA, USA), with 2 nm bandwidth.

SERS spectra were measured with a MultiRAM FT-Raman spectrometer (Bruker, Germany) working in back scattering configuration, with excitation wavelength at 1064 nm. The resolution was set to 4 cm${}^{-1}$ and incident power was kept at 250 mW. The spectra were obtained by averaging 1000 scans.

### 2.3. Computational Details

Ab initio calculations within the DFT framework were performed with the Gaussian09 suite of programs [58] using the B3LYP exchange and correlation functional [59,60,61] along with the 6-311++G(d,p) basis set for all atoms but silver, which was described with the LANL2TZ basis set [62,63,64,65]. The molecular structure and the vibrational frequency calculations were carried out imposing a very tight criterion and an improved grid in the numerical evaluation of the integrals, INTEGRAL (GRID = 199974). It was verified that all the vibrational frequencies are real, confirming that the optimized structures are true minima [66].

The Raman and SERS intensities were obtained from the computed Raman activities, using the relationship [67,68,69]:

(1) $$I_{i}=\frac{f{\left(\nu_{0}-\nu_{i}\right)}^{4}A_{i}}{\nu_{i}\left(1-e^{\frac{-hc\nu_{i}}{k_{B}T}}\right)}$$

where $I_{i}$ and $A_{i}$ are the intensity and activity of the vibrational mode *i*, respectively; $\nu_{0}$ is the exciting frequency (in cm${}^{-1}$); $\nu_{i}$ is the vibrational frequency of the *i*th normal mode (in cm${}^{-1}$); *h*, *c*, and $k_{B}$ are fundamental constants; and *f* is a normalization factor for all peak intensities. The calculated spectra were reported by assigning to each normal mode a Lorentzian shape with a 25 cm${}^{-1}$ full width at half-maximum. The vibrational frequencies were scaled by a 0.981 factor, in agreement with previous calculation on anthraquinone [16].

## 3. Results and Discussion

**Figure 1:**
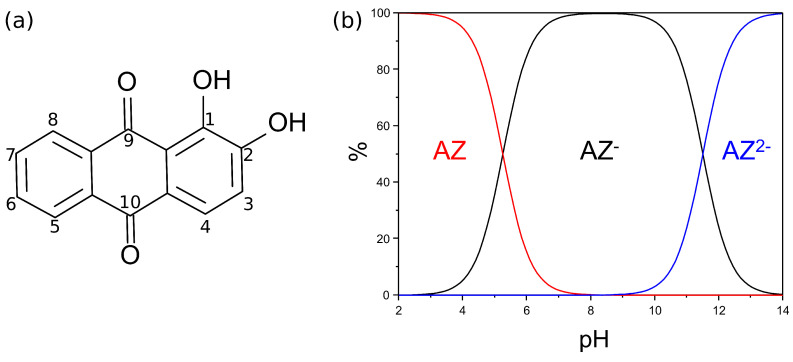
(**a**) Alizarin skeletal with atom labels. (**b**) Distribution diagram for alizarin species as a function of pH, for the AZ/AZ${}^{-}$/AZ${}^{2-}$ system. The two pK of AZ are 5.25 and 11.5, respectively.

Alizarin is an anthraquinone dye with the molecular structure shown in Figure 1a. Since we are interested in interpreting the results of SERS spectra of AZ at alkaline pH (between 10 and 11), a first important information is to state the existence in the pH range of the different species of this dye in solution. Cañamares et al. [18] established that the pK of alizarin are 5.25 and 11.5 to give rise to the AZ${}^{-}$ (monoanionic) and AZ${}^{2-}$ (dianionic) species, respectively. Moreover, Cañamares et al. [18] reported that the initial deprotonation of AZ${}^{-}$ is attributable to the loss of the H${}^{+}$ ion by the oxygen atom bound to C2 (see Figure 1a for atom labeling).

Figure 1b shows the distribution diagram of the AZ, AZ${}^{-}$, and AZ${}^{2-}$ species as a function of pH. In the experimental conditions of the SERS experiments, with a pH between 10 and 11, both AZ${}^{-}$ and AZ${}^{2-}$ species are present in the solution. This is the first useful information to select suitable models for the calculation of the SERS spectra.

**Figure 2:**
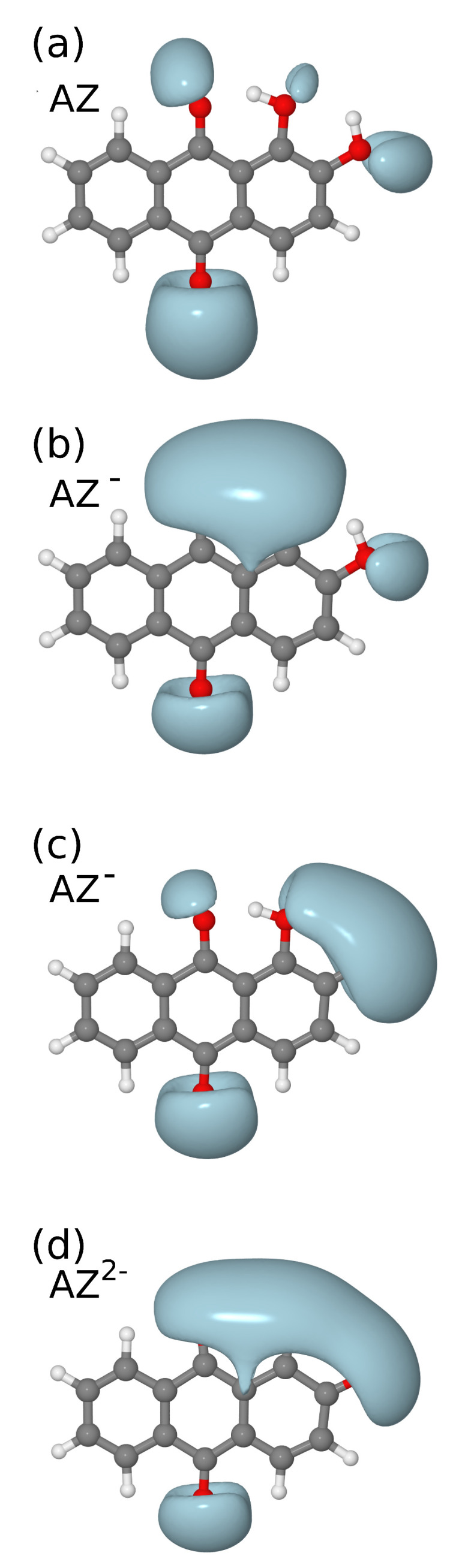
Electrostatic potential for: (**a**) AZ; (**b**) AZ${}^{-}$ with deprotonation on oxygen atom bound to C1; (**c**) AZ${}^{-}$ with deprotonation on oxygen atom bound to C2; and (**d**) AZ${}^{2-}$. The C1 and C2 atom labels are reported in Figure 1a.

The analysis of the electrostatic potential (shown in Figure 2) for AZ, AZ${}^{-}$ (with deprotonation on oxygen atoms bound to C1 or C2) and AZ${}^{2-}$ suggests that, if oxygen deprotonation occurs on C1, AZ${}^{-}$ can interact as a bidentate ligand with metal substrate similarly to the models adopted in previous studies to interpret the AZ SERS spectra [17,18,19]. For completeness and to ascertain the correctness of the subsequent model of AZ${}^{-}$ with silver, the DFT calculations for both the structure optimizations and the vibrational frequencies were carried out considering also the deprotonation on C2. Finally, the calculation was performed also for AZ${}^{2-}$ species, and, for the first time, the SERS spectra were computed also for the interaction of AZ${}^{2-}$ with a silver cluster, as discussed in the following.

Regarding the substrate, it has been observed and it is now accepted that, during the silver reduction process for the formation of silver nanoparticles, the following reactions take place for the formation of charged species (Ag${}_{3}$)${}^{+}$ and (Ag${}_{4}$)${}^{2+}$ [40,41]:

(2) $${\mathrm{Ag}}^{+}+e^{-}⟶\mathrm{Ag}$$

(3) $${\mathrm{Ag}}^{+}+\mathrm{Ag}⟶{\left({\mathrm{Ag}}_{2}\right)}^{+}$$

(4) $${\left({\mathrm{Ag}}_{2}\right)}^{+}+\mathrm{Ag}⟶{\left({\mathrm{Ag}}_{3}\right)}^{+}$$

(5) $$2{\left({\mathrm{Ag}}_{2}\right)}^{+}⟶{\left({\mathrm{Ag}}_{4}\right)}^{2+}$$

The choice to use these silver cluster to model the substrate has been further corroborate by experimental finding [38,70]. It has been established that silver substrates have a sizable amount of Ag(I) on the surface, which acts as an active site on which the organic molecule can interact [38,70]. Therefore, the modeling of the metal surface was carried out considering the two clusters (Ag${}_{3}$)${}^{+}$ and (Ag${}_{4}$)${}^{2+}$, which, although not allowing to take into account the effect of the surface responsible for the electromagnetic enhancement of the Raman signals, proved to be suitable for reproducing the relative intensities and the observed shifts due to the chemical interaction in a whole series of molecules [38,42,49,50,53,54,71].

The accuracy of the calculations was initially verified by comparing the calculated vibrational frequencies of AZ with the assignment of Pagliai et al. [16], which is based on DFT calculations at B3LYP/6-31G(d) level; the assignment is an excellent starting point for the subsequent interpretation and discussion of the SERS spectra. The comparison is reported in Table 1 and it shows a good agreement of the computed vibrational frequencies with those of the assignment [16].

**Figure 3:**
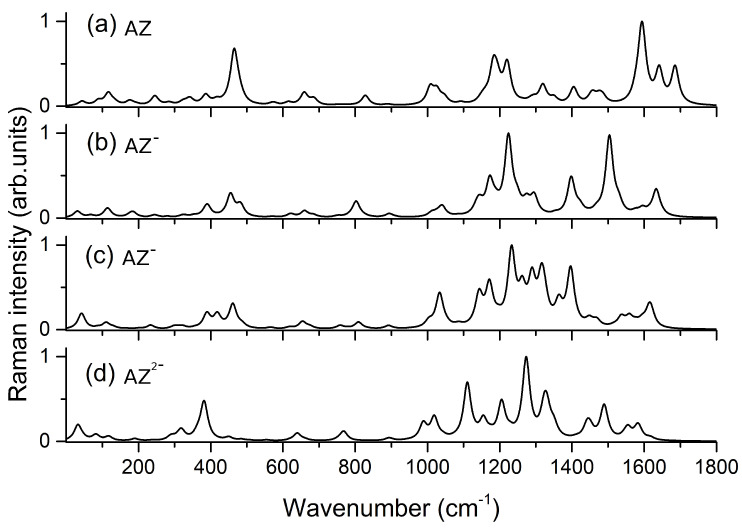
Computed Raman spectra for: (**a**) AZ; (**b**) AZ${}^{-}$ (deprotonation on the oxygen atom bound to C1); (**c**) AZ${}^{-}$ (deprotonation on the oxygen atom bound to C2); and (**d**) AZ${}^{2-}$.

The calculations were also extended to different forms of alizarin, as shown by the vibrational frequencies and Raman activities reported in Tables S1–S4 and by the simulated Raman spectra in Figure 3. The spectra of the different species of alizarin in solution at the different pH values are a useful result to rationalize the variations which are observed experimentally by interaction of the dye with the metal substrate.

**Figure 4:**
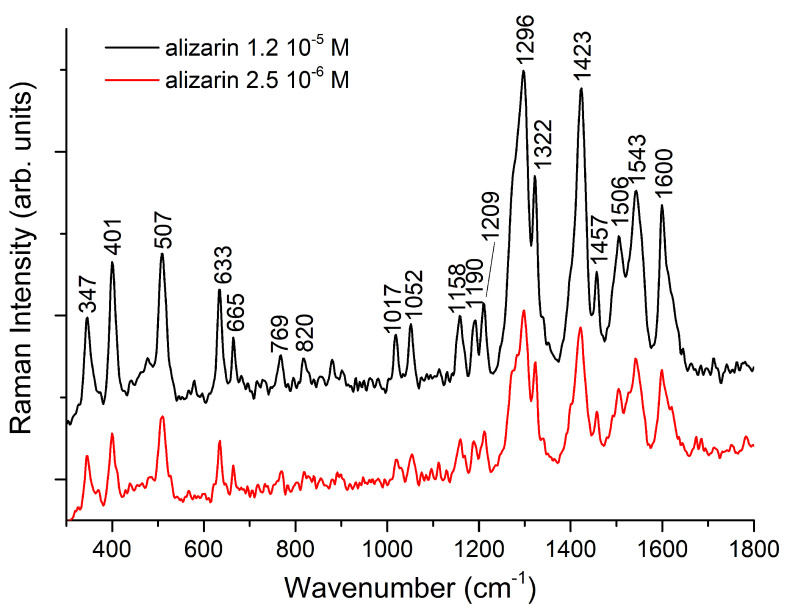
SERS spectra of alizarin. Excitation wavelength 1064 nm, 250 mW, 1000 scans. AgNP background has been subtracted.

As stated above, the experimental SERS spectrum (shown in Figure 4) was measured at pH between 10 and 11 where AZ${}^{-}$ and AZ${}^{2-}$ coexist in solution. To simplify the analysis of SERS spectra and reduce the number of models to be used in the calculations, it was decided to perform the optimization and vibrational frequency calculations only for the AZ${}^{-}$ and AZ${}^{2-}$ species interacting with (Ag${}_{3}$)${}^{+}$ and (Ag${}_{4}$)${}^{2+}$ silver clusters, respectively. Therefore, the calculations were performed for globally neutral complexes.

**Figure 5:**
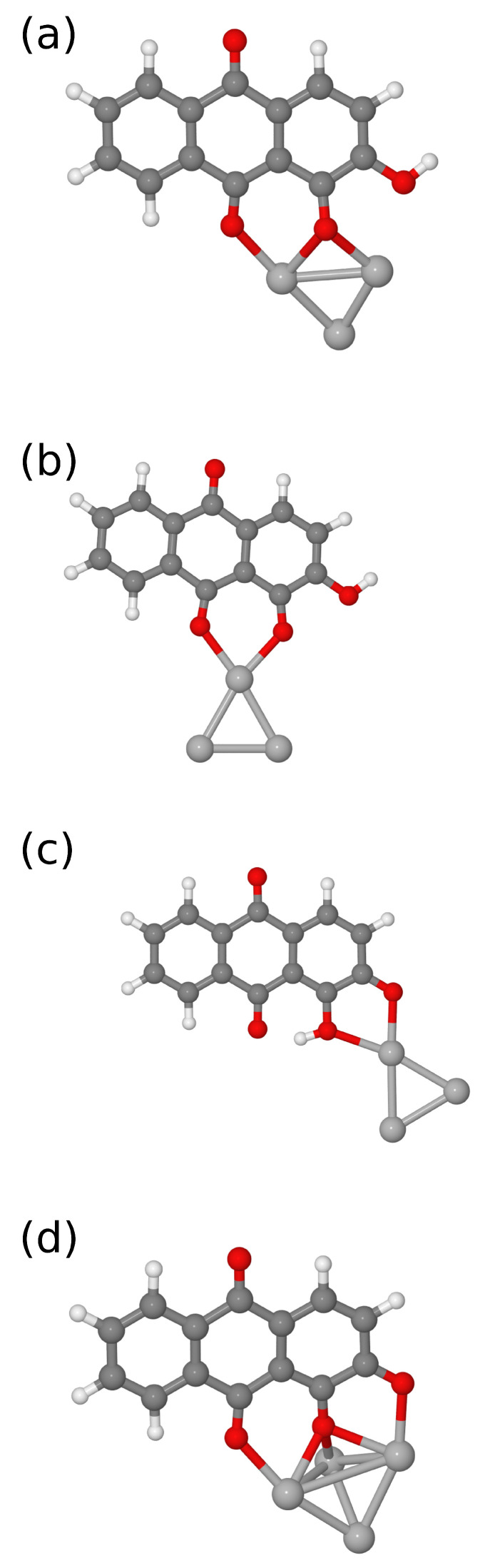
Optimized molecular structure of alizarin interacting as: AZ${}^{-}$ with (Ag${}_{3}$)${}^{+}$ cluster (**a**–**c**); and AZ${}^{2-}$ with (Ag${}_{4}$)${}^{2+}$ for (**d**).

**Figure 6:**
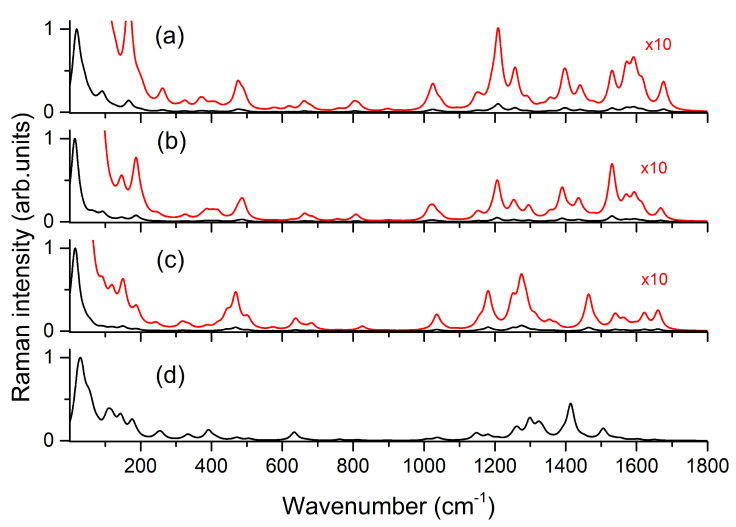
Black lines are the computed SERS spectra of alizarin interacting with silver clusters. The labels refer to models shown in Figure 5. Red lines are the 10× magnification of the respective black spectra.

The structures of the different optimized models are reported in Figure 5, while the simulated SERS spectra are shown in Figure 6. Depending on whether the dye is in the form AZ${}^{-}$ or AZ${}^{2-}$ and on the interaction of (Ag${}_{3}$)${}^{+}$ with the oxygen atoms, the calculated SERS spectra turn out to be different from each other. The different interaction of the (Ag${}_{3}$)${}^{+}$ cluster also leads to differences in simulated SERS spectra, but this can be an interesting aspect to more correctly describe adsorption geometry through a careful comparison between simulated and measured SERS spectra.

**Figure 7:**
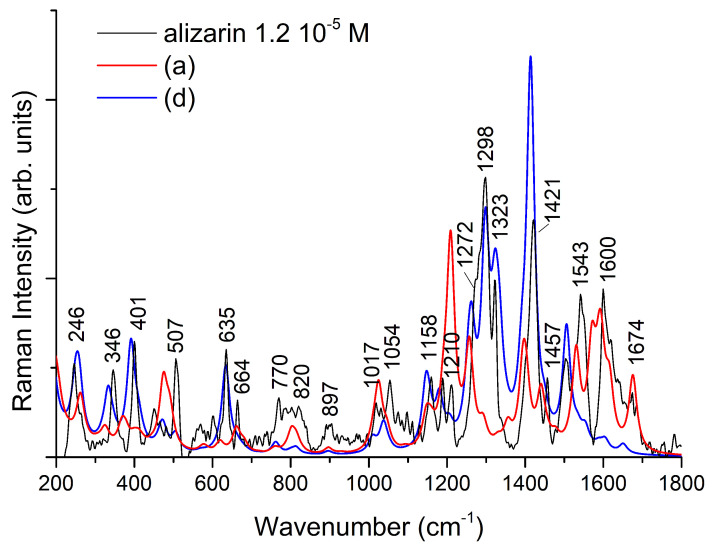
Comparison of the measured SERS spectra of alizarin with those calculated for the **a** and **d** models shown in Figure 5. The calculated intensities were uniformly scaled to match the experiment.

All calculated frequencies are reported in Tables S5–S7. Similar to the results reported by Cañamares et al. [18], the comparison between measured and simulated spectra in the frequency range between 0 and 1800 cm${}^{-1}$ can take advantage of the calculations on two different models. In the present study, the best agreement can be obtained by considering the simulated spectra for **a** and **d** models shown in Figure 5. In the latter, the interaction with silver involves oxygen atoms bound to C1, C2, and C9. This structure has not been taken into account in previous works to interpret the experimental spectra, but the calculated SERS spectrum faithfully reproduces most of the experimental features measured by some authors at alkaline pH [18,19,24,72]. However, a comparison between the measured SERS spectrum with those obtained by DFT calculations, as shown in Figure 7, suggests that this is not the only contribution to be taken into account. On the basis of the comparison, it is possible to note that the measured SERS spectrum can be faithfully reproduced considering in addition to the AZ${}^{2-}$/(Ag${}_{4}$)${}^{2+}$ model also the contribution of AZ${}^{-}$ species interacting with (Ag${}_{3}$)${}^{+}$, confirming the importance to consider all the alizarin species (AZ${}^{-}$ and AZ${}^{2-}$) present in solution. These results also provide important information on the adsorption geometry of the dye with metal substrate. In fact, the models adopted to simulate the SERS spectrum involve the interaction of alizarin through oxygen atoms bound to both C1 and C9 with the silver surface [17,18].

## 4. Conclusions

The SERS spectrum of alizarin at pH between 10 and 11 was re-interpreted on the basis of ab initio calculations in the framework of the density functional theory at the level B3LYP/6-311++G(d,p)/LanL2TZ. The active sites of the silver nanoparticles were modeled considering the clusters of (Ag${}_{3}$)${}^{+}$ and (Ag${}_{4}$)${}^{2+}$, which have already proved effective in reproducing the relative intensities in simulated SERS spectra of other systems [38,42,49,50,53,54,71]. The SERS spectrum of alizarin, due to the interaction between the two species of the dye in alkaline solution, AZ${}^{-}$ and AZ${}^{2-}$, with silver nanoparticles was faithfully simulated with calculations on AZ${}^{-}$/(Ag${}_{3}$)${}^{+}$ and AZ${}^{2-}$/(Ag${}_{4}$)${}^{2+}$ to model the coexistence of these two species in solution. In fact, the final spectrum was achieved by properly adding the results of the calculations on either the complexes. The DFT calculations provided useful information on the adsorption geometry of alizarin on silver nanostructured surfaces, which allowed faithfully reproducing the experimental SERS spectrum.

Further insights on the adsorption geometry of AZ on silver surface could be theoretically achieved by performing calculations modeling the substrate with as slab or increasing the number of atoms [73,74,75,76,77,78,79,80,81,82,83] and experimentally by using other techniques such as X-ray photoelectron spectroscopy (XPS) and Near Edge X-Ray Absorption Fine Structure (NEXAFS) spectroscopy [38,39,43,84].

## Figures and Tables

**Table 1:** Comparison of alizarin infrared and Raman vibrational frequencies computed at B3LYP/6-31G(d) [16] and B3LYP/6-311++G(d,p) level of theory (present work). The experimental infrared and Raman frequencies (in cm${}^{-1}$) and assignment were taken from Cyranski et al. [21] and Pagliai et al. [16], respectively. The normal modes were labeled as ν for stretching, δ for in plane bending or deformation, γ for out of plane bending or deformation, and sh for shoulder.

	sym	B3LYP/6-31G(d) [16]	B3LYP/6-311++G(d,p)	IR	Raman	Assignment [16]
1	$a^{\prime }$	47	43			$\gamma_{\mathrm{OH}}+\gamma_{C-O}+\gamma_{C=O}+\gamma_{ring}$
2	$a^{"}$	93	89			$\gamma_{\mathrm{OH}}+\gamma_{C-O}+\gamma_{C=O}+\gamma_{ring}$
3	$a^{"}$	123	116			$\gamma_{\mathrm{OH}}+\gamma_{C-O}+\gamma_{C=O}+\gamma_{ring}$
4	$a^{"}$	139	134			$\gamma_{\mathrm{OH}}+\gamma_{C-O}+\gamma_{C=O}+\gamma_{ring}$
5	$a^{"}$	178	176		182	$\gamma_{\mathrm{OH}}+\gamma_{C-O}+\gamma_{C=O}+\gamma_{ring}+\gamma_{\mathrm{CH}}$
6	$a^{{}^{\prime }}$	192	192		193	$\delta_{\mathrm{OH}}+\delta_{C-O}+\delta_{C=O}+\delta_{ring}+\delta_{\mathrm{CH}}$
7	$a^{\prime }$	250	245		261	$\gamma_{\mathrm{CH}}+\gamma_{C=O}+\gamma_{ring}$
8	$a^{{}^{\prime }}$	283	285		296	$\delta_{\mathrm{OH}}+\delta_{C-O}+\delta_{C=O}+\delta_{ring}+\delta_{\mathrm{CH}}$
9	$a^{{}^{\prime }}$	320	321			$\delta_{\mathrm{OH}}+\delta_{C-O}+\delta_{C=O}+\delta_{ring}+\delta_{\mathrm{CH}}$
10	$a^{"}$	329	325			$\gamma_{\mathrm{OH}}+\gamma_{C-O}+\gamma_{ring}+\gamma_{\mathrm{CH}}$
11	$a^{{}^{\prime }}$	345	342		347	$\delta_{\mathrm{OH}}+\delta_{C-O}+\delta_{C=O}+\delta_{\mathrm{CH}}$
12	$a^{{}^{\prime }}$	385	386		392	$\delta_{\mathrm{OH}}+\delta_{C=O}+\delta_{\mathrm{CH}}$
13	$a^{{}^{\prime }}$	417	416		419	$\delta_{\mathrm{OH}}+\delta_{C-O}+\delta_{C=O}+\delta_{ring}+\delta_{\mathrm{CH}}$
14	$a^{"}$	417	417	419		$\gamma_{\mathrm{CH}}+\gamma_{ring}$
15	$a^{"}$	444	440			$\gamma_{\mathrm{CH}}+\gamma_{ring}$
16	$a^{\prime }$	462	453			$\delta_{\mathrm{OH}}+\delta_{C=O}+\delta_{ring}$
17	$a^{{}^{\prime }}$	475	465		470	$\delta_{\mathrm{OH}}+\delta_{C-O}+\delta_{C=O}+\delta_{ring}+\delta_{\mathrm{CH}}$
18	$a^{{}^{\prime }}$	478	477	486	486	$\gamma_{\mathrm{OH}}+\gamma_{\mathrm{CH}}$
19	$a^{"}$	499	488	499	501	$\gamma_{\mathrm{OH}}+\gamma_{\mathrm{CH}}$
20	$a^{"}$	562	564			$\gamma_{\mathrm{CH}}+\gamma_{ring}$
21	$a^{{}^{\prime }}$	568	573	579		$\delta_{ring}+\delta_{\mathrm{OH}}$
22	$a^{{}^{\prime }}$	608	615	620	620	$\delta_{\mathrm{CH}}+\delta_{ring}$
23	$a^{{}^{\prime }}$	653	659		646	$\delta_{\mathrm{CH}}$
24	$a^{"}$	656	666	660	662	$\gamma_{\mathrm{CH}}+\gamma_{ring}$
25	$a^{{}^{\prime }}$	678	686	678	682	$\delta_{ring}+\delta_{\mathrm{OH}}+\delta_{\mathrm{CH}}$
26	$a^{"}$	684	691	700		$\gamma_{\mathrm{CH}}+\gamma_{\mathrm{OH}}$
27	$a^{"}$	712	719	712	710	$\gamma_{\mathrm{CH}}+\gamma_{\mathrm{OH}}$
28	$a^{{}^{\prime }}$	744	753	736		$\delta_{ring}+\delta_{\mathrm{OH}}$
29	$a^{"}$	768	767	748		$\gamma_{ring}+\gamma_{\mathrm{OH}}$
30	$a^{"}$	779	775	765	763	$\gamma_{\mathrm{OH}}$
31	$a^{"}$	788	794	792	795	$\gamma_{\mathrm{CH}}+\gamma_{\mathrm{OH}}$
32	$a^{{}^{\prime }}$	825	828	828	830	$\delta_{ring}+\delta_{\mathrm{OH}}+\delta_{\mathrm{CH}}$
33	$a^{"}$	840	846	848		$\gamma_{\mathrm{CH}}$
34	$a^{{}^{\prime }}$	881	889	858		$\delta_{ring}+\delta_{\mathrm{CH}}$
35	$a^{"}$	896	899	895	895	$\gamma_{\mathrm{CH}}$
36	$a^{"}$	945	959	931		$\gamma_{\mathrm{CH}}$
37	$a^{"}$	965	984	955	960	$\gamma_{\mathrm{CH}}$
38	$a^{"}$	985	1000	972		$\gamma_{\mathrm{CH}}$
39	$a^{{}^{\prime }}$	1006	1007	1012	1012	$\delta_{\mathrm{OH}}+\delta_{\mathrm{CH}}+\delta_{ring}$
40	$a^{{}^{\prime }}$	1024	1026	1031	1030	$\delta_{\mathrm{OH}}+\delta_{\mathrm{CH}}$
41	$a^{{}^{\prime }}$	1043	1046	1048	1048	$\delta_{\mathrm{OH}}+\delta_{\mathrm{CH}}$
42	$a^{{}^{\prime }}$	1085	1092	1102	1102	$\delta_{\mathrm{OH}}+\delta_{\mathrm{CH}}$
43	$a^{{}^{\prime }}$	1144	1149	1150sh	1150	$\delta_{\mathrm{OH}}+\delta_{\mathrm{CH}}$
44	$a^{{}^{\prime }}$	1156	1160	1160	1164	$\delta_{\mathrm{CH}}$
45	$a^{{}^{\prime }}$	1179	1182	1175		$\delta_{\mathrm{OH}}+\delta_{\mathrm{CH}}$
46	$a^{{}^{\prime }}$	1193	1193	1198	1191	$\delta_{\mathrm{OH}}+\delta_{\mathrm{CH}}$
47	$a^{{}^{\prime }}$	1227	1221	1220	1216	$\delta_{\mathrm{OH}}+\delta_{\mathrm{CH}}$
48	$a^{{}^{\prime }}$	1259	1261	1266	1270	$\delta_{\mathrm{OH}}+\delta_{\mathrm{CH}}$
49	$a^{{}^{\prime }}$	1284	1282	1295	1295sh	$\delta_{\mathrm{CH}}$
50	$a^{{}^{\prime }}$	1298	1293	1300sh	1300sh	$\delta_{\mathrm{OH}}+\delta_{\mathrm{CH}}$
51	$a^{{}^{\prime }}$	1327	1319		1330	$\delta_{\mathrm{OH}}+\delta_{\mathrm{CH}}$
52	$a^{{}^{\prime }}$	1337	1332	1332	1332	$\delta_{\mathrm{OH}}+\delta_{\mathrm{CH}}+\delta_{ring}$
53	$a^{{}^{\prime }}$	1359	1350	1350	1350	$\delta_{\mathrm{OH}}+\delta_{\mathrm{CH}}$
54	$a^{{}^{\prime }}$	1415	1405	1398	1399	$\delta_{\mathrm{OH}}+\delta_{\mathrm{CH}}$
55	$a^{{}^{\prime }}$	1454	1454	1429		$\delta_{\mathrm{CH}}$
56	$a^{{}^{\prime }}$	1465	1458	1452	1451	$\delta_{\mathrm{OH}}+\delta_{\mathrm{CH}}$
57	$a^{{}^{\prime }}$	1475	1476	1465	1463	$\delta_{\mathrm{CH}}+\delta_{C=O}$
58	$a^{{}^{\prime }}$	1484	1486	1477	1481	$\delta_{\mathrm{OH}}+\delta_{\mathrm{CH}}$
59	$a^{{}^{\prime }}$	1578	1578	1571	1574	$\nu_{ring}+\delta_{\mathrm{CH}}$
60	$a^{{}^{\prime }}$	1594	1592		1587	$\nu_{ring}+\delta_{\mathrm{OH}}+\delta_{\mathrm{CH}}$
61	$a^{{}^{\prime }}$	1597	1595	1589		$\nu_{ring}+\delta_{\mathrm{OH}}+\delta_{\mathrm{CH}}$
62	$a^{{}^{\prime }}$	1602	1601		1595	$\nu_{ring}+\delta_{\mathrm{OH}}$
63	$a^{{}^{\prime }}$	1647	1641	1633	1632	$\nu_{C=O}+\delta_{\mathrm{OH}}$
64	$a^{{}^{\prime }}$	1692	1685	1663	1658	$\delta_{\mathrm{OH}}+\nu_{C=O}$
65	$a^{{}^{\prime }}$	3098	3111			$\nu_{\mathrm{CH}}$
66	$a^{{}^{\prime }}$	3112	3125			$\nu_{\mathrm{CH}}$
67	$a^{{}^{\prime }}$	3120	3130			$\nu_{\mathrm{CH}}$
68	$a^{{}^{\prime }}$	3130	3141			$\nu_{\mathrm{OH}}$
69	$a^{{}^{\prime }}$	3131	3143			$\nu_{\mathrm{CH}}$
70	$a^{{}^{\prime }}$	3133	3147			$\nu_{\mathrm{CH}}$
71	$a^{{}^{\prime }}$	3136	3224			$\nu_{\mathrm{CH}}$
72	$a^{{}^{\prime }}$	3568	3684			$\nu_{\mathrm{OH}}$

## Supplementary Materials

The following are available online at https://www.mdpi.com/article/10.3390/nano11040860/s1: Tables S1–S4: vibrational frequencies and Raman activities of simulated spectra shown in Figure 3; Tables S5–S8: vibrational frequencies and Raman activities of simulated spectra shown in Figure 6.
