# Supplementary material for: Adsorption Geometry of Alizarin on Silver Nanoparticles: A Computational and Spectroscopic Study
Source: Nanomaterials (Basel). 2021 Mar 27;11(4):860. doi: 10.3390/nano11040860 (PMC8065795; doi:10.3390/nano11040860)
Supplement: Supplementary file 1 [file nanomaterials-11-00860-s001.pdf]

# Supplementary Material for Adsorption Geometry of Alizarin on Silver Nanoparticles: a Computational and Spectroscopic Study

Cristina Gellini,* Marina Macchiagodena, and Marco Pagliai*

*Dipartimento di Chimica "Ugo Schiff", Università degli Studi di Firenze, Via della Lastruccia 3, 50019 Sesto Fiorentino, Italy*

E-mail: cristina.gellini@unifi.it; marco.pagliai@unifi.it

Table S1: Calculated and scaled (0.981) normal mode frequencies ($cm^1$), infrared intensities (IR), and Raman activities (Raman) of alizarin (AZ) at B3LYP/6-311++G(d,p) level of theory.

| | sym | frequencies | scaled | IR | Raman |
|---|---|---|---|---|---|
| 1 | $a^{"}$ | 44.14 | 43.30 | 2.851 | 0.0595 |
| 2 | $a^{"}$ | 90.41 | 88.70 | 0.4488 | 0.246 |
| 3 | $a^{"}$ | 118.73 | 116.48 | 1.1292 | 1.0575 |
| 4 | $a^{"}$ | 136.32 | 133.73 | 0.0047 | 0.2759 |
| 5 | $a^{"}$ | 179.00 | 175.60 | 0.0843 | 0.8 |
| 6 | $a^{'}$ | 195.58 | 191.86 | 0.5979 | 0.2548 |
| 7 | $a^{"}$ | 250.06 | 245.31 | 0.2788 | 2.9736 |
| 8 | $a^{'}$ | 290.09 | 284.58 | 1.3719 | 1.0208 |
| 9 | $a^{'}$ | 327.13 | 320.91 | 0.2414 | 0.572 |
| 10 | $a^{"}$ | 331.98 | 325.67 | 0.6504 | 1.1007 |
| 11 | $a^{'}$ | 348.72 | 342.09 | 7.1482 | 3.3885 |
| 12 | $a^{'}$ | 393.43 | 385.95 | 5.6536 | 6.1083 |
| 13 | $a^{'}$ | 424.45 | 416.39 | 21.2545 | 2.7208 |
| 14 | $a^{"}$ | 424.98 | 416.91 | 0.2631 | 0.0801 |
| 15 | $a^{"}$ | 448.69 | 440.17 | 17.1066 | 0.223 |
| 16 | $a^{"}$ | 461.80 | 453.02 | 102.871 | 0.9913 |
| 17 | $a^{'}$ | 473.89 | 464.89 | 0.2902 | 44.1586 |
| 18 | $a^{'}$ | 486.64 | 477.39 | 12.9854 | 6.7051 |
| 19 | $a^{"}$ | 497.55 | 488.10 | 14.4248 | 1.3458 |
| 20 | $a^{"}$ | 574.94 | 564.01 | 0.3491 | 0.0961 |
| 21 | $a^{'}$ | 584.13 | 573.04 | 6.1636 | 3.1088 |
| 22 | $a^{'}$ | 626.70 | 614.79 | 13.7296 | 3.6714 |

Continued on next page

|  | sym | frequencies | scaled | IR | Raman |
|---|---|---|---|---|---|
| 23 | $a^{'}$ | 671.61 | 658.85 | 16.0475 | 17.2867 |
| 24 | $a^{"}$ | 679.07 | 666.16 | 2.748 | 0.0539 |
| 25 | $a^{'}$ | 698.79 | 685.52 | 3.3622 | 9.3186 |
| 26 | $a^{"}$ | 704.36 | 690.98 | 0 | 0.0281 |
| 27 | $a^{"}$ | 733.37 | 719.44 | 65.674 | 0.0272 |
| 28 | $a^{'}$ | 767.18 | 752.60 | 6.0587 | 1.4809 |
| 29 | $a^{"}$ | 781.52 | 766.67 | 5.5618 | 0.0073 |
| 30 | $a^{"}$ | 789.83 | 774.82 | 91.1058 | 0.9505 |
| 31 | $a^{"}$ | 809.33 | 793.96 | 3.3673 | 0.5505 |
| 32 | $a^{'}$ | 843.81 | 827.77 | 42.8969 | 19.8975 |
| 33 | $a^{"}$ | 862.44 | 846.06 | 22.6535 | 0.0147 |
| 34 | $a^{'}$ | 906.70 | 889.47 | 8.0215 | 2.7556 |
| 35 | $a^{"}$ | 916.24 | 898.83 | 0.119 | 0.0056 |
| 36 | $a^{"}$ | 977.65 | 959.07 | 0.0235 | 0.3715 |
| 37 | $a^{"}$ | 1002.83 | 983.78 | 1.802 | 0.0015 |
| 38 | $a^{"}$ | 1019.37 | 1000.00 | 0.0728 | 0.0713 |
| 39 | $a^{'}$ | 1026.99 | 1007.47 | 58.1284 | 43.6985 |
| 40 | $a^{'}$ | 1045.79 | 1025.92 | 12.2582 | 33.7089 |
| 41 | $a^{'}$ | 1066.53 | 1046.26 | 8.6225 | 17.6141 |
| 42 | $a^{'}$ | 1113.34 | 1092.19 | 0.7614 | 5.6626 |
| 43 | $a^{'}$ | 1171.48 | 1149.22 | 1.6341 | 12.7164 |
| 44 | $a^{'}$ | 1182.32 | 1159.85 | 14.819 | 15.4238 |
| 45 | $a^{'}$ | 1205.26 | 1182.36 | 38.6992 | 115.446 |
| 46 | $a^{'}$ | 1216.27 | 1193.16 | 75.66 | 58.3112 |
| 47 | $a^{'}$ | 1244.28 | 1220.64 | 21.0452 | 138.201 |

Continued on next page

| | sym | frequencies | scaled | IR | Raman |
|---|---|---|---|---|---|
| 48 | $a^{'}$ | 1285.69 | 1261.26 | 92.8893 | 4.4616 |
| 49 | $a^{'}$ | 1307.20 | 1282.36 | 981.339 | 6.1249 |
| 50 | $a^{'}$ | 1317.97 | 1292.93 | 84.4023 | 16.9421 |
| 51 | $a^{'}$ | 1344.86 | 1319.31 | 98.709 | 74.5836 |
| 52 | $a^{'}$ | 1357.81 | 1332.01 | 46.4758 | 2.0527 |
| 53 | $a^{'}$ | 1376.18 | 1350.03 | 161.081 | 24.3862 |
| 54 | $a^{'}$ | 1431.95 | 1404.74 | 2.5511 | 75.1676 |
| 55 | $a^{'}$ | 1481.71 | 1453.56 | 33.024 | 20.7032 |
| 56 | $a^{'}$ | 1486.11 | 1457.87 | 392.353 | 31.5884 |
| 57 | $a^{'}$ | 1504.29 | 1475.71 | 1.5085 | 33.4658 |
| 58 | $a^{'}$ | 1514.60 | 1485.83 | 6.5626 | 27.8741 |
| 59 | $a^{'}$ | 1608.01 | 1577.46 | 6.6202 | 43.565 |
| 60 | $a^{'}$ | 1622.42 | 1591.60 | 142.884 | 127.476 |
| 61 | $a^{'}$ | 1625.42 | 1594.53 | 23.5782 | 282.478 |
| 62 | $a^{'}$ | 1631.62 | 1600.62 | 45.0388 | 44.2299 |
| 63 | $a^{'}$ | 1673.11 | 1641.33 | 168.551 | 190.936 |
| 64 | $a^{'}$ | 1717.99 | 1685.35 | 173.787 | 226.781 |
| 65 | $a^{'}$ | 3171.57 | 3111.31 | 3.2176 | 79.5562 |
| 66 | $a^{'}$ | 3185.91 | 3125.38 | 10.6523 | 204.84 |
| 67 | $a^{'}$ | 3191.08 | 3130.45 | 2.7183 | 123.036 |
| 68 | $a^{'}$ | 3201.51 | 3140.68 | 0.9182 | 45.3003 |
| 69 | $a^{'}$ | 3204.27 | 3143.39 | 10.2783 | 204.014 |
| 70 | $a^{'}$ | 3207.48 | 3146.54 | 1.5462 | 149.579 |
| 71 | $a^{'}$ | 3286.48 | 3224.04 | 288.442 | 316.46 |
| 72 | $a^{'}$ | 3755.57 | 3684.21 | 129.611 | 137.057 |

Table S2: Calculated and scaled (0.981) normal mode frequencies ($cm^1$), infrared intensities (IR), and Raman activities (Raman) of alizarin anion ($AZ^-$; oxygen atom bound to C1) at B3LYP/6-311++G(d,p) level of theory.

| | sym | frequencies | scaled | IR | Raman |
|---|---|---|---|---|---|
| 1 | $a^"$ | 31.18 | 30.59 | 3.8961 | 0.0952 |
| 2 | $a^"$ | 68.79 | 67.48 | 1.2237 | 0.1324 |
| 3 | $a^"$ | 114.81 | 112.63 | 0.0288 | 0.9421 |
| 4 | $a^"$ | 118.53 | 116.28 | 1.2284 | 0.7696 |
| 5 | $a^"$ | 179.22 | 175.81 | 1.0344 | 0.6799 |
| 6 | $a^{'}$ | 188.50 | 184.92 | 1.1011 | 2.015 |
| 7 | $a^"$ | 249.19 | 244.46 | 0.2241 | 1.6838 |
| 8 | $a^{'}$ | 285.68 | 280.25 | 0.0141 | 0.7197 |
| 9 | $a^{'}$ | 328.21 | 321.97 | 8.2527 | 1.8033 |
| 10 | $a^"$ | 336.75 | 330.35 | 0.1273 | 0.7733 |
| 11 | $a^{'}$ | 360.70 | 353.85 | 16.5302 | 1.7495 |
| 12 | $a^{'}$ | 392.14 | 384.69 | 16.6803 | 3.4891 |
| 13 | $a^{'}$ | 398.44 | 390.87 | 12.4168 | 14.3167 |
| 14 | $a^"$ | 426.97 | 418.86 | 0.0882 | 0.2197 |
| 15 | $a^"$ | 446.92 | 438.43 | 0.4519 | 0.822 |
| 16 | $a^{'}$ | 463.59 | 454.79 | 10.6986 | 38.6951 |
| 17 | $a^"$ | 484.80 | 475.59 | 0.9441 | 1.1471 |
| 18 | $a^{'}$ | 491.83 | 482.49 | 8.4769 | 20.8947 |
| 19 | $a^{'}$ | 581.20 | 570.15 | 8.7639 | 1.8176 |
| 20 | $a^"$ | 587.71 | 576.54 | 0.1304 | 0.1817 |
| 21 | $a^{'}$ | 633.34 | 621.30 | 12.1194 | 9.3967 |
| 22 | $a^{'}$ | 672.50 | 659.72 | 16.7615 | 18.6607 |

Continued on next page

| | sym | frequencies | scaled | IR | Raman |
|---|---|---|---|---|---|
| 23 | $a^{"}$ | 683.12 | 670.15 | 0.4118 | 0.041 |
| 24 | $a^{'}$ | 697.12 | 683.87 | 0.5067 | 6.7338 |
| 25 | $a^{"}$ | 726.13 | 712.34 | 63.6441 | 0.0601 |
| 26 | $a^{"}$ | 758.51 | 744.10 | 20.8848 | 0.3024 |
| 27 | $a^{'}$ | 767.31 | 752.73 | 15.7565 | 4.7423 |
| 28 | $a^{"}$ | 790.84 | 775.81 | 0.3639 | 0.5088 |
| 29 | $a^{"}$ | 804.39 | 789.11 | 5.7316 | 3.1319 |
| 30 | $a^{'}$ | 818.36 | 802.81 | 22.7283 | 62.4171 |
| 31 | $a^{"}$ | 833.85 | 818.01 | 49.9212 | 0.6037 |
| 32 | $a^{"}$ | 841.39 | 825.40 | 64.4045 | 2.0521 |
| 33 | $a^{"}$ | 899.43 | 882.35 | 0.1133 | 0.0449 |
| 34 | $a^{'}$ | 910.87 | 893.57 | 2.0557 | 15.9749 |
| 35 | $a^{"}$ | 925.11 | 907.53 | 0.185 | 1.9292 |
| 36 | $a^{"}$ | 987.61 | 968.85 | 1.9158 | 0.0394 |
| 37 | $a^{"}$ | 997.41 | 978.46 | 0.7701 | 0.0161 |
| 38 | $a^{'}$ | 1031.03 | 1011.44 | 103.801 | 20.0399 |
| 39 | $a^{'}$ | 1044.74 | 1024.89 | 3.3757 | 12.9018 |
| 40 | $a^{'}$ | 1060.40 | 1040.25 | 0.0669 | 60.0929 |
| 41 | $a^{'}$ | 1105.88 | 1084.86 | 2.603 | 4.5584 |
| 42 | $a^{'}$ | 1157.79 | 1135.80 | 2.4642 | 39.0581 |
| 43 | $a^{'}$ | 1166.21 | 1144.06 | 4.0128 | 73.6021 |
| 44 | $a^{'}$ | 1195.24 | 1172.53 | 26.1115 | 226.522 |
| 45 | $a^{'}$ | 1212.20 | 1189.17 | 21.3985 | 51.8313 |
| 46 | $a^{'}$ | 1247.83 | 1224.13 | 99.4757 | 582.968 |
| 47 | $a^{'}$ | 1272.12 | 1247.95 | 66.9965 | 90.9234 |

Continued on next page

| | sym | frequencies | scaled | IR | Raman |
|---|---|---|---|---|---|
| 48 | $a^{'}$ | 1299.28 | 1274.59 | 116.796 | 93.6388 |
| 49 | $a^{'}$ | 1320.59 | 1295.50 | 819.115 | 146.61 |
| 50 | $a^{'}$ | 1348.71 | 1323.09 | 1.8248 | 1.0948 |
| 51 | $a^{'}$ | 1380.90 | 1354.66 | 74.8788 | 15.1621 |
| 52 | $a^{'}$ | 1425.03 | 1397.95 | 40.7999 | 352.63 |
| 53 | $a^{'}$ | 1449.62 | 1422.08 | 159.444 | 67.137 |
| 54 | $a^{'}$ | 1474.24 | 1446.23 | 16.0163 | 6.685 |
| 55 | $a^{'}$ | 1496.95 | 1468.51 | 1.8154 | 38.8283 |
| 56 | $a^{'}$ | 1532.94 | 1503.82 | 775.33 | 841.518 |
| 57 | $a^{'}$ | 1559.23 | 1529.61 | 25.9943 | 109.009 |
| 58 | $a^{'}$ | 1606.35 | 1575.83 | 4.2272 | 38.3643 |
| 59 | $a^{'}$ | 1626.14 | 1595.24 | 102.846 | 71.593 |
| 60 | $a^{'}$ | 1646.32 | 1615.04 | 3.5622 | 22.9616 |
| 61 | $a^{'}$ | 1664.78 | 1633.15 | 106.938 | 323.598 |
| 62 | $a^{'}$ | 1680.80 | 1648.86 | 405.008 | 14.2514 |
| 63 | $a^{'}$ | 3143.06 | 3083.35 | 10.7184 | 104.458 |
| 64 | $a^{'}$ | 3155.06 | 3095.11 | 42.7974 | 220.682 |
| 65 | $a^{'}$ | 3161.66 | 3101.59 | 47.9196 | 322.527 |
| 66 | $a^{'}$ | 3188.82 | 3128.23 | 6.784 | 49.3152 |
| 67 | $a^{'}$ | 3191.35 | 3130.72 | 25.9394 | 174.241 |
| 68 | $a^{'}$ | 3193.40 | 3132.72 | 5.7425 | 144.694 |
| 69 | $a^{'}$ | 3259.83 | 3197.89 | 167.853 | 191.564 |

Table S3: Calculated and scaled (0.981) normal mode frequencies ($cm^1$), infrared intensities (IR), and Raman activities (Raman) of alizarin anion ($AZ^-$; oxygen atom bound to C2) at B3LYP/6-311++G(d,p) level of theory.

| | sym | frequencies | scaled | IR | Raman |
|---|---|---|---|---|---|
| 1 | $a^"$ | 32.58 | 31.96 | 1.2902 | 0.6277 |
| 2 | $a^"$ | 46.21 | 45.33 | 0.0158 | 0.1256 |
| 3 | $a^"$ | 83.47 | 81.88 | 6.248 | 1.3956 |
| 4 | $a^"$ | 119.44 | 117.17 | 0.2223 | 1.972 |
| 5 | $a^"$ | 169.53 | 166.31 | 3.1068 | 0.0457 |
| 6 | $a^{'}$ | 193.17 | 189.50 | 0.5355 | 2.3154 |
| 7 | $a^"$ | 240.76 | 236.19 | 0.3131 | 0.7893 |
| 8 | $a^{'}$ | 295.73 | 290.11 | 6.3298 | 8.993 |
| 9 | $a^"$ | 316.88 | 310.86 | 0.1525 | 4.8761 |
| 10 | $a^{'}$ | 324.97 | 318.80 | 17.0677 | 22.8551 |
| 11 | $a^{'}$ | 369.57 | 362.55 | 3.5472 | 14.0155 |
| 12 | $a^{'}$ | 387.78 | 380.41 | 9.37 | 59.0981 |
| 13 | $a^{'}$ | 389.97 | 382.56 | 0.1928 | 69.3055 |
| 14 | $a^"$ | 429.81 | 421.64 | 0.7012 | 0.1122 |
| 15 | $a^"$ | 443.73 | 435.30 | 0.1044 | 0.3981 |
| 16 | $a^{'}$ | 458.98 | 450.26 | 14.0791 | 13.1729 |
| 17 | $a^{'}$ | 494.84 | 485.44 | 22.6782 | 5.6087 |
| 18 | $a^"$ | 516.93 | 507.11 | 0.001 | 2.6982 |
| 19 | $?_a$ | 564.68 | 553.96 | 1.3429 | 0.4296 |
| 20 | $?_a$ | 564.77 | 554.04 | 6.9985 | 4.9125 |
| 21 | $a^{'}$ | 630.70 | 618.72 | 12.5797 | 4.3666 |
| 22 | $a^{'}$ | 651.92 | 639.54 | 1.0371 | 52.746 |

Continued on next page

| | sym | frequencies | scaled | IR | Raman |
|---|---|---|---|---|---|
| 23 | $a^{"}$ | 661.66 | 649.08 | 2.2602 | 0.1605 |
| 24 | $a^{'}$ | 673.58 | 660.78 | 13.2368 | 8.6184 |
| 25 | $a^{"}$ | 707.52 | 694.08 | 79.193 | 0.2982 |
| 26 | $a^{"}$ | 754.45 | 740.11 | 4.3611 | 1.0215 |
| 27 | $a^{'}$ | 767.64 | 753.05 | 10.5448 | 7.3731 |
| 28 | $a^{"}$ | 775.57 | 760.83 | 27.4762 | 0.5352 |
| 29 | $a^{"}$ | 780.43 | 765.60 | 6.7229 | 11.284 |
| 30 | $a^{'}$ | 783.33 | 768.44 | 4.6632 | 76.3495 |
| 31 | $a^{"}$ | 838.26 | 822.33 | 5.6799 | 0.7663 |
| 32 | $a^{"}$ | 853.85 | 837.63 | 0.0784 | 0.3514 |
| 33 | $a^{'}$ | 910.69 | 893.38 | 3.5163 | 30.2864 |
| 34 | $a^{"}$ | 956.86 | 938.68 | 0.0402 | 0.1859 |
| 35 | $a^{"}$ | 960.96 | 942.70 | 1.3158 | 0.3577 |
| 36 | $a^{"}$ | 972.32 | 953.85 | 3.2398 | 1.3919 |
| 37 | $a^{'}$ | 1007.43 | 988.29 | 21.5091 | 212.389 |
| 38 | $a^{'}$ | 1038.42 | 1018.69 | 54.6952 | 303.68 |
| 39 | $a^{'}$ | 1062.46 | 1042.27 | 48.3218 | 26.8255 |
| 40 | $a^{'}$ | 1096.04 | 1075.21 | 5.2231 | 6.9097 |
| 41 | $a^{'}$ | 1131.76 | 1110.25 | 33.6968 | 886.615 |
| 42 | $a^{'}$ | 1152.30 | 1130.40 | 6.0397 | 11.0982 |
| 43 | $a^{'}$ | 1177.02 | 1154.66 | 66.7028 | 294.216 |
| 44 | $a^{'}$ | 1196.68 | 1173.95 | 13.6863 | 52.8464 |
| 45 | $a^{'}$ | 1228.64 | 1205.30 | 157.253 | 638.981 |
| 46 | $a^{'}$ | 1253.30 | 1229.49 | 48.8139 | 75.6257 |
| 47 | $a^{'}$ | 1297.89 | 1273.23 | 41.9029 | 1580.73 |

Continued on next page

| | sym | frequencies | scaled | IR | Raman |
|---|---|---|---|---|---|
| 48 | $a^{'}$ | 1348.92 | 1323.29 | 58.8135 | 544.974 |
| 49 | $a^{'}$ | 1357.01 | 1331.23 | 37.0754 | 454.275 |
| 50 | $a^{'}$ | 1376.04 | 1349.89 | 194.241 | 247.26 |
| 51 | $a^{'}$ | 1439.15 | 1411.81 | 229.262 | 28.9484 |
| 52 | $a^{'}$ | 1469.66 | 1441.73 | 151.888 | 294.789 |
| 53 | $a^{'}$ | 1478.01 | 1449.93 | 18.1084 | 219.217 |
| 54 | $a^{'}$ | 1517.35 | 1488.52 | 933.753 | 696.004 |
| 55 | $a^{'}$ | 1521.21 | 1492.31 | 73.5935 | 198.648 |
| 56 | $a^{'}$ | 1576.29 | 1546.34 | 377.021 | 57.5633 |
| 57 | $a^{'}$ | 1585.06 | 1554.95 | 988.443 | 296.351 |
| 58 | $a^{'}$ | 1610.65 | 1580.05 | 131.937 | 148.389 |
| 59 | $a^{'}$ | 1615.38 | 1584.69 | 23.0591 | 297.187 |
| 60 | $a^{'}$ | 1648.26 | 1616.94 | 356.582 | 71.2014 |
| 61 | $a^{'}$ | 3095.84 | 3037.02 | 136.155 | 599.965 |
| 62 | $a^{'}$ | 3102.90 | 3043.95 | 25.7331 | 167.228 |
| 63 | $a^{'}$ | 3126.19 | 3066.79 | 128.883 | 469.437 |
| 64 | $a^{'}$ | 3150.42 | 3090.56 | 31.7905 | 80.4318 |
| 65 | $a^{'}$ | 3168.92 | 3108.71 | 15.508 | 39.8514 |
| 66 | $a^{'}$ | 3171.14 | 3110.89 | 48.3127 | 232.33 |

Table S4: Calculated and scaled (0.981) normal mode frequencies ($cm^1$), infrared intensities (IR), and Raman activities (Raman) of alizarin dianion ($AZ^{2-}$) at B3LYP/6-311++G(d,p) level of theory.

| | sym | frequencies | scaled | IR | Raman |
|---|---|---|---|---|---|
| 1 | $a^{"}$ | 32.58 | 31.96 | 1.2902 | 0.6277 |
| 2 | $a^{"}$ | 46.21 | 45.33 | 0.0158 | 0.1256 |
| 3 | $a^{"}$ | 83.47 | 81.88 | 6.248 | 1.3956 |
| 4 | $a^{"}$ | 119.44 | 117.17 | 0.2223 | 1.972 |
| 5 | $a^{"}$ | 169.53 | 166.31 | 3.1068 | 0.0457 |
| 6 | $a^{'}$ | 193.17 | 189.50 | 0.5355 | 2.3154 |
| 7 | $a^{"}$ | 240.76 | 236.19 | 0.3131 | 0.7893 |
| 8 | $a^{'}$ | 295.73 | 290.11 | 6.3298 | 8.993 |
| 9 | $a^{"}$ | 316.88 | 310.86 | 0.1525 | 4.8761 |
| 10 | $a^{'}$ | 324.97 | 318.80 | 17.0677 | 22.8551 |
| 11 | $a^{'}$ | 369.57 | 362.55 | 3.5472 | 14.0155 |
| 12 | $a^{'}$ | 387.78 | 380.41 | 9.37 | 59.0981 |
| 13 | $a^{'}$ | 389.97 | 382.56 | 0.1928 | 69.3055 |
| 14 | $a^{"}$ | 429.81 | 421.64 | 0.7012 | 0.1122 |
| 15 | $a^{"}$ | 443.73 | 435.30 | 0.1044 | 0.3981 |
| 16 | $a^{'}$ | 458.98 | 450.26 | 14.0791 | 13.1729 |
| 17 | $a^{'}$ | 494.84 | 485.44 | 22.6782 | 5.6087 |
| 18 | $a^{"}$ | 516.93 | 507.11 | 0.001 | 2.6982 |
| 19 | $?_a$ | 564.68 | 553.96 | 1.3429 | 0.4296 |
| 20 | $?_a$ | 564.77 | 554.04 | 6.9985 | 4.9125 |
| 21 | $a^{'}$ | 630.70 | 618.72 | 12.5797 | 4.3666 |
| 22 | $a^{'}$ | 651.92 | 639.54 | 1.0371 | 52.746 |

Continued on next page

| | sym | frequencies | scaled | IR | Raman |
|---|---|---|---|---|---|
| 23 | $a^{"}$ | 661.66 | 649.08 | 2.2602 | 0.1605 |
| 24 | $a^{'}$ | 673.58 | 660.78 | 13.2368 | 8.6184 |
| 25 | $a^{"}$ | 707.52 | 694.08 | 79.193 | 0.2982 |
| 26 | $a^{"}$ | 754.45 | 740.11 | 4.3611 | 1.0215 |
| 27 | $a^{'}$ | 767.64 | 753.05 | 10.5448 | 7.3731 |
| 28 | $a^{"}$ | 775.57 | 760.83 | 27.4762 | 0.5352 |
| 29 | $a^{"}$ | 780.43 | 765.60 | 6.7229 | 11.284 |
| 30 | $a^{'}$ | 783.33 | 768.44 | 4.6632 | 76.3495 |
| 31 | $a^{"}$ | 838.26 | 822.33 | 5.6799 | 0.7663 |
| 32 | $a^{"}$ | 853.85 | 837.63 | 0.0784 | 0.3514 |
| 33 | $a^{'}$ | 910.69 | 893.38 | 3.5163 | 30.2864 |
| 34 | $a^{"}$ | 956.86 | 938.68 | 0.0402 | 0.1859 |
| 35 | $a^{"}$ | 960.96 | 942.70 | 1.3158 | 0.3577 |
| 36 | $a^{"}$ | 972.32 | 953.85 | 3.2398 | 1.3919 |
| 37 | $a^{'}$ | 1007.43 | 988.29 | 21.5091 | 212.389 |
| 38 | $a^{'}$ | 1038.42 | 1018.69 | 54.6952 | 303.68 |
| 39 | $a^{'}$ | 1062.46 | 1042.27 | 48.3218 | 26.8255 |
| 40 | $a^{'}$ | 1096.04 | 1075.21 | 5.2231 | 6.9097 |
| 41 | $a^{'}$ | 1131.76 | 1110.25 | 33.6968 | 886.615 |
| 42 | $a^{'}$ | 1152.30 | 1130.40 | 6.0397 | 11.0982 |
| 43 | $a^{'}$ | 1177.02 | 1154.66 | 66.7028 | 294.216 |
| 44 | $a^{'}$ | 1196.68 | 1173.95 | 13.6863 | 52.8464 |
| 45 | $a^{'}$ | 1228.64 | 1205.30 | 157.253 | 638.981 |
| 46 | $a^{'}$ | 1253.30 | 1229.49 | 48.8139 | 75.6257 |
| 47 | $a^{'}$ | 1297.89 | 1273.23 | 41.9029 | 1580.73 |

Continued on next page

| | sym | frequencies | scaled | IR | Raman |
|---|---|---|---|---|---|
| 48 | $a^{'}$ | 1348.92 | 1323.29 | 58.8135 | 544.974 |
| 49 | $a^{'}$ | 1357.01 | 1331.23 | 37.0754 | 454.275 |
| 50 | $a^{'}$ | 1376.04 | 1349.89 | 194.241 | 247.26 |
| 51 | $a^{'}$ | 1439.15 | 1411.81 | 229.262 | 28.9484 |
| 52 | $a^{'}$ | 1469.66 | 1441.73 | 151.888 | 294.789 |
| 53 | $a^{'}$ | 1478.01 | 1449.93 | 18.1084 | 219.217 |
| 54 | $a^{'}$ | 1517.35 | 1488.52 | 933.753 | 696.004 |
| 55 | $a^{'}$ | 1521.21 | 1492.31 | 73.5935 | 198.648 |
| 56 | $a^{'}$ | 1576.29 | 1546.34 | 377.021 | 57.5633 |
| 57 | $a^{'}$ | 1585.06 | 1554.95 | 988.443 | 296.351 |
| 58 | $a^{'}$ | 1610.65 | 1580.05 | 131.937 | 148.389 |
| 59 | $a^{'}$ | 1615.38 | 1584.69 | 23.0591 | 297.187 |
| 60 | $a^{'}$ | 1648.26 | 1616.94 | 356.582 | 71.2014 |
| 61 | $a^{'}$ | 3095.84 | 3037.02 | 136.155 | 599.965 |
| 62 | $a^{'}$ | 3102.90 | 3043.95 | 25.7331 | 167.228 |
| 63 | $a^{'}$ | 3126.19 | 3066.79 | 128.883 | 469.437 |
| 64 | $a^{'}$ | 3150.42 | 3090.56 | 31.7905 | 80.4318 |
| 65 | $a^{'}$ | 3168.92 | 3108.71 | 15.508 | 39.8514 |
| 66 | $a^{'}$ | 3171.14 | 3110.89 | 48.3127 | 232.33 |

Table S5: Calculated and scaled (0.981) normal mode frequencies ($cm^1$), infrared intensities (IR), and Raman activities (Raman) of model (a) in Figure 5 of the manuscript at B3LYP/6-311++G(d,p) level of theory. Silver has been treated with LANL2TZ basis set.

| | sym | frequencies | scaled | IR | Raman |
|---|---|---|---|---|---|
| 1 | *a* | 18.35 | 18.01 | 0.0652 | 2.6365 |
| 2 | *a* | 24.49 | 24.03 | 0.8992 | 1.1039 |
| 3 | *a* | 39.04 | 38.29 | 1.2776 | 1.86 |
| 4 | *a* | 42.07 | 41.27 | 2.1223 | 1.621 |
| 5 | *a* | 58.13 | 57.02 | 2.8907 | 2.5261 |
| 6 | *a* | 74.98 | 73.56 | 2.6813 | 1.0301 |
| 7 | *a* | 84.29 | 82.69 | 0.3029 | 2.4485 |
| 8 | *a* | 94.69 | 92.89 | 2.7264 | 12.7651 |
| 9 | *a* | 112.99 | 110.85 | 0.869 | 1.9125 |
| 10 | *a* | 120.75 | 118.46 | 0.527 | 1.7094 |
| 11 | *a* | 131.17 | 128.68 | 2.1643 | 3.0196 |
| 12 | *a* | 166.94 | 163.77 | 1.8476 | 8.0364 |
| 13 | *a* | 170.77 | 167.53 | 8.7197 | 18.9664 |
| 14 | *a* | 197.13 | 193.39 | 16.6179 | 3.2863 |
| 15 | *a* | 207.72 | 203.78 | 17.3033 | 3.1981 |
| 16 | *a* | 267.10 | 262.02 | 21.0958 | 9.6216 |
| 17 | *a* | 310.51 | 304.61 | 5.0021 | 0.7518 |
| 18 | *a* | 328.17 | 321.93 | 2.7973 | 2.1402 |
| 19 | *a* | 332.91 | 326.58 | 22.4375 | 3.2177 |
| 20 | *a* | 374.91 | 367.79 | 63.2867 | 7.2352 |
| 21 | *a* | 384.86 | 377.55 | 12.1699 | 5.0108 |
| 22 | *a* | 406.62 | 398.89 | 11.7007 | 3.468 |

Continued on next page

| | sym | frequencies | scaled | IR | Raman |
|---|---|---|---|---|---|
| 23 | *a* | 418.66 | 410.71 | 30.4216 | 4.1354 |
| 24 | *a* | 429.12 | 420.97 | 2.786 | 1.4949 |
| 25 | *a* | 452.92 | 444.32 | 0.9962 | 1.5403 |
| 26 | *a* | 483.44 | 474.26 | 3.8102 | 35.4882 |
| 27 | *a* | 497.41 | 487.96 | 1.0405 | 2.1656 |
| 28 | *a* | 500.64 | 491.12 | 10.6825 | 17.0268 |
| 29 | *a* | 585.49 | 574.37 | 8.3652 | 2.9503 |
| 30 | *a* | 592.23 | 580.98 | 1.9914 | 2.3888 |
| 31 | *a* | 631.15 | 619.16 | 5.9558 | 8.4003 |
| 32 | *a* | 674.05 | 661.24 | 19.3637 | 20.7449 |
| 33 | *a* | 691.11 | 677.98 | 0.8357 | 3.8976 |
| 34 | *a* | 695.23 | 682.02 | 4.0326 | 4.5881 |
| 35 | *a* | 731.68 | 717.77 | 69.2943 | 0.7954 |
| 36 | *a* | 767.80 | 753.21 | 20.8955 | 1.4761 |
| 37 | *a* | 775.96 | 761.22 | 17.3501 | 4.8443 |
| 38 | *a* | 801.66 | 786.43 | 2.3629 | 1.3715 |
| 39 | *a* | 817.57 | 802.04 | 6.5187 | 23.6217 |
| 40 | *a* | 830.40 | 814.62 | 27.691 | 14.8757 |
| 41 | *a* | 844.64 | 828.59 | 29.9333 | 1.3871 |
| 42 | *a* | 912.96 | 895.61 | 3.12 | 0.6289 |
| 43 | *a* | 914.11 | 896.74 | 12.1274 | 7.6571 |
| 44 | *a* | 947.23 | 929.23 | 0.166 | 1.8455 |
| 45 | *a* | 998.78 | 979.80 | 1.7225 | 0.0755 |
| 46 | *a* | 1013.26 | 994.01 | 0.0949 | 0.1907 |
| 47 | *a* | 1031.00 | 1011.41 | 17.6485 | 19.4309 |

Continued on next page

| | sym | frequencies | scaled | IR | Raman |
|---|---|---|---|---|---|
| 48 | $a$ | 1044.66 | 1024.82 | 75.068 | 104.695 |
| 49 | $a$ | 1066.00 | 1045.75 | 3.13 | 29.7348 |
| 50 | $a$ | 1114.16 | 1093.00 | 1.0683 | 5.0188 |
| 51 | $a$ | 1168.89 | 1146.68 | 104.989 | 51.2979 |
| 52 | $a$ | 1172.50 | 1150.22 | 204.22 | 8.7139 |
| 53 | $a$ | 1181.75 | 1159.30 | 117.369 | 29.2332 |
| 54 | $a$ | 1214.40 | 1191.32 | 10.5703 | 73.495 |
| 55 | $a$ | 1232.75 | 1209.33 | 128.237 | 434.615 |
| 56 | $a$ | 1269.89 | 1245.76 | 258.567 | 7.7156 |
| 57 | $a$ | 1281.65 | 1257.30 | 266.244 | 223.72 |
| 58 | $a$ | 1317.40 | 1292.37 | 220.643 | 52.2164 |
| 59 | $a$ | 1356.01 | 1330.25 | 3.8361 | 17.9391 |
| 60 | $a$ | 1381.75 | 1355.49 | 87.2856 | 55.2471 |
| 61 | $a$ | 1421.46 | 1394.46 | 61.8626 | 184.657 |
| 62 | $a$ | 1429.42 | 1402.26 | 260.377 | 132.486 |
| 63 | $a$ | 1468.55 | 1440.65 | 101.025 | 154.383 |
| 64 | $a$ | 1483.58 | 1455.39 | 70.3501 | 19.3025 |
| 65 | $a$ | 1506.09 | 1477.48 | 1.0667 | 31.7676 |
| 66 | $a$ | 1560.23 | 1530.59 | 307.245 | 299.039 |
| 67 | $a$ | 1601.32 | 1570.90 | 231.059 | 304.348 |
| 68 | $a$ | 1621.01 | 1590.21 | 20.2938 | 204.249 |
| 69 | $a$ | 1626.37 | 1595.47 | 182.535 | 172.724 |
| 70 | $a$ | 1647.27 | 1615.97 | 213.33 | 193.376 |
| 71 | $a$ | 1708.26 | 1675.80 | 234.211 | 279.333 |
| 72 | $a$ | 3140.66 | 3080.98 | 26.1814 | 199.001 |

Continued on next page

| | sym | frequencies | scaled | IR | Raman |
|---|---|---|---|---|---|
| 73 | $a$ | 3167.08 | 3106.91 | 3.5369 | 83.7609 |
| 74 | $a$ | 3182.30 | 3121.84 | 17.3317 | 267.45 |
| 75 | $a$ | 3200.33 | 3139.52 | 6.4474 | 144.149 |
| 76 | $a$ | 3207.35 | 3146.41 | 2.7028 | 58.6527 |
| 77 | $a$ | 3207.68 | 3146.74 | 6.2694 | 169.336 |
| 78 | $a$ | 3825.88 | 3753.19 | 104.295 | 258.704 |

Table S6: Calculated and scaled (0.981) normal mode frequencies ($cm^1$), infrared intensities (IR), and Raman activities (Raman) of model (b) in Figure 5 of the manuscript at B3LYP/6-311++G(d,p) level of theory. Silver has been treated with LANL2TZ basis set.

| | sym | frequencies | scaled | IR | Raman |
|---|---|---|---|---|---|
| 1 | *a* | 12.47 | 12.23 | 0.0998 | 0.0604 |
| 2 | *a* | 14.53 | 14.26 | 0.1394 | 2.8514 |
| 3 | *a* | 16.78 | 16.46 | 0.3653 | 0.094 |
| 4 | *a* | 32.32 | 31.71 | 3.0416 | 0.4235 |
| 5 | *a* | 64.04 | 62.82 | 1.1138 | 2.4307 |
| 6 | *a* | 73.66 | 72.26 | 6.8602 | 1.1097 |
| 7 | *a* | 74.25 | 72.84 | 6.7305 | 0.6897 |
| 8 | *a* | 95.09 | 93.28 | 1.3981 | 9.1134 |
| 9 | *a* | 106.57 | 104.55 | 0.0447 | 0.3874 |
| 10 | *a* | 119.78 | 117.50 | 0.0597 | 0.891 |
| 11 | *a* | 137.00 | 134.40 | 0.416 | 0.5357 |
| 12 | *a* | 149.51 | 146.67 | 10.475 | 8.2625 |
| 13 | *a* | 181.00 | 177.56 | 0.9515 | 0.7692 |
| 14 | *a* | 190.75 | 187.12 | 20.4034 | 24.3521 |
| 15 | *a* | 210.17 | 206.17 | 2.6045 | 2.7532 |
| 16 | *a* | 251.84 | 247.06 | 0.0238 | 2.3867 |
| 17 | *a* | 310.23 | 304.34 | 6.9332 | 0.1225 |
| 18 | *a* | 319.54 | 313.47 | 26.963 | 0.3372 |
| 19 | *a* | 332.57 | 326.25 | 0.466 | 4.6485 |
| 20 | *a* | 378.98 | 371.78 | 63.7779 | 3.2758 |
| 21 | *a* | 393.31 | 385.84 | 9.0632 | 10.9753 |
| 22 | *a* | 411.02 | 403.21 | 1.9336 | 7.8202 |

Continued on next page

| | sym | frequencies | scaled | IR | Raman |
|---|---|---|---|---|---|
| 23 | *a* | 426.08 | 417.99 | 40.9319 | 11.0955 |
| 24 | *a* | 428.12 | 419.99 | 0.3396 | 0.04 |
| 25 | *a* | 455.12 | 446.47 | 1.2593 | 0.855 |
| 26 | *a* | 484.11 | 474.91 | 1.133 | 15.2139 |
| 27 | *a* | 492.77 | 483.41 | 0.2436 | 1.4927 |
| 28 | *a* | 497.65 | 488.19 | 5.6198 | 38.142 |
| 29 | *a* | 585.25 | 574.13 | 0.0183 | 0.3266 |
| 30 | *a* | 591.35 | 580.11 | 14.2809 | 1.3373 |
| 31 | *a* | 637.37 | 625.26 | 2.1459 | 2.8764 |
| 32 | *a* | 675.91 | 663.07 | 17.7417 | 22.6257 |
| 33 | *a* | 688.55 | 675.47 | 1.0185 | 0.0025 |
| 34 | *a* | 698.40 | 685.13 | 9.1535 | 10.752 |
| 35 | *a* | 730.20 | 716.32 | 65.6815 | 0.0322 |
| 36 | *a* | 767.68 | 753.09 | 24.0882 | 0.4624 |
| 37 | *a* | 770.57 | 755.93 | 23.9687 | 6.4787 |
| 38 | *a* | 798.67 | 783.49 | 1.6759 | 0.3857 |
| 39 | *a* | 816.48 | 800.96 | 0.1362 | 1.4832 |
| 40 | *a* | 823.86 | 808.21 | 16.0605 | 28.3779 |
| 41 | *a* | 840.98 | 825.00 | 32.436 | 0.351 |
| 42 | *a* | 910.11 | 892.82 | 0.2142 | 0.0055 |
| 43 | *a* | 915.42 | 898.03 | 7.3464 | 5.4722 |
| 44 | *a* | 943.33 | 925.40 | 0.2915 | 1.616 |
| 45 | *a* | 996.92 | 977.98 | 1.4595 | 0.0055 |
| 46 | *a* | 1010.33 | 991.13 | 0.1569 | 0.0957 |
| 47 | *a* | 1034.05 | 1014.40 | 23.819 | 59.5216 |

Continued on next page

| | sym | frequencies | scaled | IR | Raman |
|---|---|---|---|---|---|
| 48 | $a$ | 1046.90 | 1027.01 | 128.693 | 64.2269 |
| 49 | $a$ | 1065.60 | 1045.35 | 6.3322 | 24.7475 |
| 50 | $a$ | 1114.51 | 1093.33 | 3.3841 | 3.6315 |
| 51 | $a$ | 1170.75 | 1148.50 | 32.7281 | 10.3641 |
| 52 | $a$ | 1172.96 | 1150.67 | 135.387 | 41.0343 |
| 53 | $a$ | 1182.13 | 1159.67 | 198.651 | 14.5471 |
| 54 | $a$ | 1214.85 | 1191.77 | 8.2359 | 22.9742 |
| 55 | $a$ | 1230.08 | 1206.71 | 109.447 | 305.654 |
| 56 | $a$ | 1277.15 | 1252.89 | 109.934 | 137.732 |
| 57 | $a$ | 1290.12 | 1265.61 | 284.271 | 24.2564 |
| 58 | $a$ | 1320.71 | 1295.62 | 243.493 | 117.529 |
| 59 | $a$ | 1355.62 | 1329.87 | 0.351 | 3.7844 |
| 60 | $a$ | 1381.49 | 1355.24 | 124.671 | 58.3513 |
| 61 | $a$ | 1416.67 | 1389.76 | 0.7757 | 305.128 |
| 62 | $a$ | 1438.81 | 1411.47 | 110.419 | 51.3319 |
| 63 | $a$ | 1464.33 | 1436.51 | 97.8854 | 197.386 |
| 64 | $a$ | 1483.18 | 1455.00 | 40.6722 | 23.1466 |
| 65 | $a$ | 1505.76 | 1477.15 | 0.3289 | 27.5351 |
| 66 | $a$ | 1560.41 | 1530.76 | 405.425 | 653.737 |
| 67 | $a$ | 1600.71 | 1570.29 | 472.527 | 209.377 |
| 68 | $a$ | 1623.91 | 1593.05 | 141.004 | 200.226 |
| 69 | $a$ | 1627.54 | 1596.62 | 107.128 | 69.4322 |
| 70 | $a$ | 1645.80 | 1614.53 | 286.966 | 142.696 |
| 71 | $a$ | 1700.36 | 1668.06 | 226.127 | 164.376 |
| 72 | $a$ | 3135.20 | 3075.63 | 38.9942 | 260.291 |

Continued on next page

| | sym | frequencies | scaled | IR | Raman |
|---|---|---|---|---|---|
| 73 | $a$ | 3163.85 | 3103.74 | 4.2314 | 85.1429 |
| 74 | $a$ | 3179.77 | 3119.35 | 20.5971 | 259.653 |
| 75 | $a$ | 3199.75 | 3138.95 | 7.217 | 140.723 |
| 76 | $a$ | 3205.21 | 3144.31 | 0.642 | 103.57 |
| 77 | $a$ | 3208.98 | 3148.01 | 8.8843 | 100.333 |
| 78 | $a$ | 3821.42 | 3748.81 | 91.978 | 305.059 |

Table S7: Calculated and scaled (0.981) normal mode frequencies ($cm^1$), infrared intensities (IR), and Raman activities (Raman) of model (c) in Figure 5 of the manuscript at B3LYP/6-311++G(d,p) level of theory. Silver has been treated with LANL2TZ basis set.

| | sym | frequencies | scaled | IR | Raman |
|---|---|---|---|---|---|
| 1 | *a* | 8.40 | 8.24 | 0.5868 | 0.1588 |
| 2 | *a* | 14.71 | 14.43 | 0.3885 | 1.4578 |
| 3 | *a* | 17.02 | 16.70 | 0.1963 | 3.2114 |
| 4 | *a* | 41.09 | 40.31 | 1.1829 | 1.9396 |
| 5 | *a* | 58.09 | 56.99 | 0.1281 | 1.8611 |
| 6 | *a* | 58.73 | 57.61 | 6.5082 | 1.0121 |
| 7 | *a* | 94.53 | 92.73 | 2.2657 | 3.5708 |
| 8 | *a* | 100.38 | 98.48 | 0.8814 | 0.0392 |
| 9 | *a* | 121.28 | 118.97 | 0.0427 | 4.8255 |
| 10 | *a* | 122.77 | 120.44 | 6.6796 | 1.5503 |
| 11 | *a* | 140.17 | 137.50 | 1.2066 | 0.238 |
| 12 | *a* | 153.16 | 150.25 | 10.1458 | 16.3199 |
| 13 | *a* | 174.48 | 171.17 | 0 | 0.7592 |
| 14 | *a* | 192.06 | 188.41 | 15.9297 | 9.7813 |
| 15 | *a* | 245.04 | 240.38 | 8.6499 | 0.6054 |
| 16 | *a* | 248.91 | 244.18 | 0.0045 | 3.3824 |
| 17 | *a* | 322.97 | 316.84 | 9.4338 | 8.7193 |
| 18 | *a* | 332.84 | 326.51 | 0.0809 | 1.4374 |
| 19 | *a* | 342.92 | 336.41 | 28.0759 | 4.9432 |
| 20 | *a* | 393.80 | 386.31 | 0.4739 | 5.4732 |
| 21 | *a* | 426.09 | 418.00 | 28.5605 | 7.417 |
| 22 | *a* | 426.22 | 418.13 | 0.4463 | 0.0355 |

Continued on next page

| | sym | frequencies | scaled | IR | Raman |
|---|---|---|---|---|---|
| 23 | $a$ | 452.14 | 443.55 | 11.5285 | 33.5083 |
| 24 | $a$ | 455.34 | 446.69 | 0.0018 | 0.3367 |
| 25 | $a$ | 477.65 | 468.58 | 1.3444 | 92.5053 |
| 26 | $a$ | 502.74 | 493.18 | 0.7116 | 0.6869 |
| 27 | $a$ | 512.03 | 502.30 | 12.1053 | 29.8241 |
| 28 | $a$ | 569.88 | 559.05 | 0.3059 | 0.0584 |
| 29 | $a$ | 584.75 | 573.64 | 12.3437 | 8.019 |
| 30 | $a$ | 649.72 | 637.37 | 11.4448 | 46.5714 |
| 31 | $a$ | 672.20 | 659.43 | 21.3664 | 10.0339 |
| 32 | $a$ | 683.80 | 670.81 | 5.5108 | 0.0995 |
| 33 | $a$ | 696.07 | 682.84 | 2.8701 | 29.6357 |
| 34 | $a$ | 730.46 | 716.58 | 31.7783 | 0.0146 |
| 35 | $a$ | 734.26 | 720.31 | 32.4535 | 0.097 |
| 36 | $a$ | 773.66 | 758.96 | 23.3823 | 3.0807 |
| 37 | $a$ | 779.74 | 764.93 | 0.3333 | 0.352 |
| 38 | $a$ | 808.30 | 792.94 | 11.6035 | 1.3835 |
| 39 | $a$ | 841.39 | 825.40 | 52.5455 | 25.7532 |
| 40 | $a$ | 854.85 | 838.61 | 46.1649 | 0.4865 |
| 41 | $a$ | 874.32 | 857.71 | 57.9665 | 1.2049 |
| 42 | $a$ | 907.80 | 890.55 | 2.056 | 3.6659 |
| 43 | $a$ | 912.60 | 895.26 | 0.5124 | 0.0158 |
| 44 | $a$ | 980.93 | 962.29 | 0.2319 | 0.6154 |
| 45 | $a$ | 998.39 | 979.43 | 1.5298 | 0.0015 |
| 46 | $a$ | 1014.74 | 995.46 | 0.2917 | 0.0293 |
| 47 | $a$ | 1024.35 | 1004.89 | 85.837 | 2.5139 |

Continued on next page

| | sym | frequencies | scaled | IR | Raman |
|---|---|---|---|---|---|
| 48 | $a$ | 1055.65 | 1035.59 | 0.4769 | 134.493 |
| 49 | $a$ | 1074.91 | 1054.49 | 1.6918 | 11.9937 |
| 50 | $a$ | 1112.19 | 1091.05 | 1.3748 | 7.1021 |
| 51 | $a$ | 1175.07 | 1152.75 | 49.6624 | 8.7562 |
| 52 | $a$ | 1178.98 | 1156.57 | 0.3803 | 65.2762 |
| 53 | $a$ | 1203.74 | 1180.87 | 23.7499 | 383.98 |
| 54 | $a$ | 1224.74 | 1201.47 | 104.997 | 27.2462 |
| 55 | $a$ | 1273.45 | 1249.25 | 69.6806 | 293.088 |
| 56 | $a$ | 1298.87 | 1274.19 | 747.872 | 505.446 |
| 57 | $a$ | 1312.77 | 1287.83 | 2.0737 | 212.126 |
| 58 | $a$ | 1338.81 | 1313.38 | 141.861 | 104.386 |
| 59 | $a$ | 1355.67 | 1329.91 | 8.3598 | 24.6846 |
| 60 | $a$ | 1379.95 | 1353.73 | 720.497 | 84.9129 |
| 61 | $a$ | 1399.03 | 1372.45 | 452.235 | 58.4363 |
| 62 | $a$ | 1481.16 | 1453.01 | 23.5954 | 14.3693 |
| 63 | $a$ | 1492.92 | 1464.56 | 594.645 | 522.135 |
| 64 | $a$ | 1503.22 | 1474.66 | 156.418 | 1.4754 |
| 65 | $a$ | 1516.41 | 1487.60 | 419.262 | 50.2126 |
| 66 | $a$ | 1569.32 | 1539.51 | 2.8825 | 233.216 |
| 67 | $a$ | 1593.86 | 1563.58 | 278.346 | 125.834 |
| 68 | $a$ | 1606.00 | 1575.49 | 134.261 | 33.4146 |
| 69 | $a$ | 1625.21 | 1594.34 | 109.531 | 58.8325 |
| 70 | $a$ | 1653.06 | 1621.66 | 63.8877 | 275.675 |
| 71 | $a$ | 1692.49 | 1660.33 | 224.703 | 360.173 |
| 72 | $a$ | 3052.98 | 2994.97 | 496.716 | 1753.16 |

Continued on next page

| | sym | frequencies | scaled | IR | Raman |
|---|---|---|---|---|---|
| 73 | $a$ | 3166.31 | 3106.15 | 4.7877 | 100.154 |
| 74 | $a$ | 3178.09 | 3117.71 | 5.5793 | 93.2175 |
| 75 | $a$ | 3181.60 | 3121.15 | 16.557 | 279.743 |
| 76 | $a$ | 3194.70 | 3134.00 | 7.0386 | 149.238 |
| 77 | $a$ | 3199.49 | 3138.70 | 1.1823 | 55.4865 |
| 78 | $a$ | 3202.03 | 3141.19 | 14.1246 | 220.846 |

Table S8: Calculated and scaled (0.981) normal mode frequencies ($cm^1$), infrared intensities (IR), and Raman activities (Raman) of model (d) in Figure 5 of the manuscript at B3LYP/6-311++G(d,p) level of theory. Silver has been treated with LANL2TZ basis set.

| | sym | frequencies | scaled | IR | Raman |
|---|---|---|---|---|---|
| 1 | *a* | 21.48 | 21.07 | 0.838 | 4.4864 |
| 2 | *a* | 27.02 | 26.51 | 0.2839 | 5.9423 |
| 3 | *a* | 34.72 | 34.06 | 0.2565 | 15.3126 |
| 4 | *a* | 51.53 | 50.55 | 0.7606 | 13.21 |
| 5 | *a* | 56.43 | 55.36 | 1.5046 | 7.9464 |
| 6 | *a* | 62.45 | 61.26 | 1.2379 | 14.982 |
| 7 | *a* | 72.18 | 70.81 | 0.8011 | 1.6814 |
| 8 | *a* | 80.37 | 78.85 | 3.7222 | 10.3147 |
| 9 | *a* | 95.67 | 93.86 | 0.1136 | 1.2565 |
| 10 | *a* | 108.15 | 106.09 | 1.1152 | 54.8805 |
| 11 | *a* | 119.70 | 117.42 | 6.9306 | 49.346 |
| 12 | *a* | 123.55 | 121.20 | 1.9183 | 4.494 |
| 13 | *a* | 126.72 | 124.31 | 2.1791 | 12.3984 |
| 14 | *a* | 146.28 | 143.50 | 0.0733 | 100.512 |
| 15 | *a* | 152.21 | 149.31 | 0.4312 | 9.4081 |
| 16 | *a* | 179.58 | 176.17 | 7.863 | 131.057 |
| 17 | *a* | 190.69 | 187.06 | 8.8344 | 19.64 |
| 18 | *a* | 253.75 | 248.93 | 1.1531 | 62.7455 |
| 19 | *a* | 263.16 | 258.16 | 5.9171 | 83.1521 |
| 20 | *a* | 323.32 | 317.18 | 12.7128 | 7.3867 |
| 21 | *a* | 339.23 | 332.78 | 9.5781 | 114.072 |
| 22 | *a* | 350.87 | 344.20 | 7.6884 | 22.8287 |

Continued on next page

| | sym | frequencies | scaled | IR | Raman |
|---|---|---|---|---|---|
| 23 | $a$ | 398.90 | 391.32 | 4.4845 | 297.073 |
| 24 | $a$ | 418.78 | 410.82 | 29.7209 | 64.6409 |
| 25 | $a$ | 430.42 | 422.24 | 2.7838 | 6.7098 |
| 26 | $a$ | 460.15 | 451.41 | 0.647 | 4.2624 |
| 27 | $a$ | 476.02 | 466.98 | 1.4585 | 26.6138 |
| 28 | $a$ | 482.02 | 472.86 | 5.4251 | 84.0392 |
| 29 | $a$ | 515.23 | 505.44 | 5.8017 | 71.3735 |
| 30 | $a$ | 524.43 | 514.47 | 1.2078 | 1.6464 |
| 31 | $a$ | 580.75 | 569.72 | 4.3062 | 8.3651 |
| 32 | $a$ | 591.68 | 580.44 | 10.1806 | 3.1067 |
| 33 | $a$ | 646.06 | 633.79 | 1.1221 | 513.696 |
| 34 | $a$ | 675.56 | 662.73 | 19.4457 | 36.9941 |
| 35 | $a$ | 689.03 | 675.94 | 6.481 | 13.2849 |
| 36 | $a$ | 697.57 | 684.31 | 3.9158 | 8.3256 |
| 37 | $a$ | 733.49 | 719.55 | 62.7122 | 2.2349 |
| 38 | $a$ | 776.02 | 761.27 | 13.1421 | 73.4755 |
| 39 | $a$ | 778.48 | 763.69 | 8.9279 | 18.4308 |
| 40 | $a$ | 802.18 | 786.93 | 8.1128 | 1.5137 |
| 41 | $a$ | 815.89 | 800.39 | 4.5907 | 23.1265 |
| 42 | $a$ | 829.78 | 814.01 | 29.4043 | 54.7626 |
| 43 | $a$ | 860.38 | 844.03 | 22.4699 | 2.377 |
| 44 | $a$ | 911.92 | 894.59 | 1.2389 | 2.0921 |
| 45 | $a$ | 913.57 | 896.22 | 14.6769 | 32.3096 |
| 46 | $a$ | 985.04 | 966.33 | 0.2743 | 1.9626 |
| 47 | $a$ | 996.65 | 977.72 | 1.3727 | 0.0721 |

Continued on next page

| | sym | frequencies | scaled | IR | Raman |
|---|---|---|---|---|---|
| 48 | $a$ | 1012.65 | 993.41 | 0.2973 | 0.3109 |
| 49 | $a$ | 1028.55 | 1009.01 | 118.591 | 163.401 |
| 50 | $a$ | 1057.59 | 1037.50 | 0.7609 | 353.643 |
| 51 | $a$ | 1070.71 | 1050.37 | 10.7831 | 32.4347 |
| 52 | $a$ | 1113.87 | 1092.71 | 1.1824 | 4.2597 |
| 53 | $a$ | 1168.42 | 1146.22 | 47.0606 | 876.248 |
| 54 | $a$ | 1177.79 | 1155.41 | 5.0793 | 239.573 |
| 55 | $a$ | 1203.33 | 1180.47 | 38.2151 | 676.604 |
| 56 | $a$ | 1229.64 | 1206.28 | 49.784 | 249.999 |
| 57 | $a$ | 1279.21 | 1254.91 | 681.624 | 774.651 |
| 58 | $a$ | 1287.56 | 1263.10 | 3.6786 | 1407.03 |
| 59 | $a$ | 1323.28 | 1298.13 | 44.555 | 3394.59 |
| 60 | $a$ | 1348.42 | 1322.80 | 236.919 | 1945.94 |
| 61 | $a$ | 1359.67 | 1333.84 | 178.592 | 1327.84 |
| 62 | $a$ | 1379.30 | 1353.09 | 360.949 | 111.564 |
| 63 | $a$ | 1418.32 | 1391.37 | 144.18 | 964.777 |
| 64 | $a$ | 1441.43 | 1414.04 | 71.2964 | 7457.78 |
| 65 | $a$ | 1478.14 | 1450.05 | 4.387 | 415.014 |
| 66 | $a$ | 1503.66 | 1475.09 | 2.7631 | 49.0297 |
| 67 | $a$ | 1535.14 | 1505.98 | 150.585 | 2621.07 |
| 68 | $a$ | 1564.20 | 1534.48 | 8.2976 | 299.05 |
| 69 | $a$ | 1584.10 | 1554.00 | 72.4531 | 422.36 |
| 70 | $a$ | 1618.82 | 1588.06 | 53.3548 | 144.957 |
| 71 | $a$ | 1635.51 | 1604.44 | 96.366 | 273.651 |
| 72 | $a$ | 1682.88 | 1650.90 | 557.755 | 243.194 |

Continued on next page

| | sym | frequencies | scaled | IR | Raman |
|---|---|---|---|---|---|
| 73 | $a$ | 3165.04 | 3104.91 | 4.3977 | 101.129 |
| 74 | $a$ | 3176.09 | 3115.74 | 9.1869 | 161.299 |
| 75 | $a$ | 3180.56 | 3120.13 | 21.8671 | 323.739 |
| 76 | $a$ | 3193.87 | 3133.19 | 7.6295 | 191.069 |
| 77 | $a$ | 3199.86 | 3139.06 | 6.4301 | 159.742 |
| 78 | $a$ | 3205.57 | 3144.66 | 11.1808 | 91.9838 |
